# Electrochemical removal of stains from paper cultural relics based on the electrode system of conductive composite hydrogel and PbO_2_

**DOI:** 10.1038/s41598-017-08907-w

**Published:** 2017-08-18

**Authors:** Xingtang Liang, Lizhen Zheng, Shirong Li, Xiaoyu Fan, Shukun Shen, Daodao Hu

**Affiliations:** 1 Engineering Research Center of Historical and Cultural Heritage Protection, Ministry of Education, School of Materials Science and Engineering, Shaanxi Normal University, Xi’an, 710062 China; 2 School of Petroleum and Chemical Engineering, Qinzhou University, Qinzhou, 535000 China

## Abstract

Constructing methods for cleaning stains on paper artworks that meet the requirements of preservation of cultural relics are still challenging. In response to this problem, a novel electrochemical cleaning method and the preparation of corresponding electrodes were proposed. For this purpose, the conductive graphene (rGO)/polyacryamide (PAM)/montmorillonite (MMT) composite hydrogel as cathode and PbO_2_-based material as anode were prepared and characterized. The electrochemical cleaning efficiencies of real sample and mimicking paper artifacts were evaluated, and the effects of the electrochemical cleaning on paper itself were detected. Based on the above experiments, the following results were obtained. The composite hydrogel with attractive mechanical properties is mainly based on the hydrogen bond interactions between PAM chains and MMT. The results of cleaning efficiency revealed that the black mildew stains together with the yellowish foxing stains were almost completely eliminated within 6 min at 8 mA/cm^2^, and various stains formed by tideline, foxing, organic dyes and drinks could be thoroughly removed at 4 mA/cm^2^ within 5 min. In addition, the proposed cleaning method has advantages in local selectivity, easy control of cleaning course, and reusability, which represents a potential utility of this approach.

## Introduction

Paper-based artifacts are one kind of popular and important historical cultural relics. As one of the primary carriers for culture and art, the paper plays a vital role in the dissemination and inheritance of information. However, paper of any kind is prone to degrade and so is inherently vulnerable, which makes ensuring the survival of paper-based artifacts difficult. Preserving paper-based artifacts is still a challenge, even for professionals^1^. Among of issues involved in the protection of paper-based artifacts, cleaning is of great significance because various contaminants can damage paper-based artifacts. For example, stains can severely damage artifacts not only in value but also in corrosion; mold growth not only deteriorates the structure of the paper itself but also permanently detracts from the visual appearance of an artifact^1, 2^. Therefore, cleaning of paper-based artifacts is necessary to decrease the further damage of contamination and improve the aesthetics of works. Additionally, cleaning of paper artifacts represents one of the most delicate operations of restoration because it is potentially invasive for the original materials as well as completely irreversible^3, 4^. One of the most important issues in cleaning process is the removal of foreign matters without affecting the original one. Namely, a controlled and selective cleaning action is advisable.

In order to clean paper-based artifacts, various approaches have been reported. Indubitably, it is the most popular that undesirable contaminants are removed by solvent dissolution. However, the cleaning of paper achieved by means of solvents (organic or water) presents several problems, such as the extensive diffusion of dissolved substances into deeper and broader areas of the artifact, the dramatic decay of the mechanical properties of paper due to swelling^5^, and adverse health effects probably caused by organic solvents. To confront these issues, thickeners such as cellulose ethers and polyacrylic acids were used to enhance the control of solvents over the cleaning process. In addition, solvent gels, mainly consist of organic solvent, water, surfactant and polymer (Carbopol), were also applied to decrease the diffusion of solvents. However, both the thickener and the solvent gel could cause intractable residuals remaining on the artwork after the cleaning^6^. To solve this problem, the cleaning based on hydrogels such as rheoreversible hydrogels^7^, nanomagnetic hydrogels^8^, rigid hydrogels^4, 9, 10^, and other peeled hydrogels^11, 12^, has been especially concerned in the past decade. During these cleaning processes, contaminants could be controllably dissolved in the hydrogel containing different detergents, and then they would be simultaneously removed when the hydrogel was peeled off from the paper in one piece^13, 14^. Owing to the high retention power and viscosity of hydrogels, the penetration of aqueous solution into the paper was significantly reduced, therefore minimizing damages^4, 6, 15^. For example, acrylamide-based nanomagnetic hydrogel and acrylamide/bisacrylamide hydrogel were loaded with microemulsions for removing synthetic adhesives^8, 11^; rigid gellan based hydrogel loaded with α-amylase and proteinase has been used for selectively cleaning starch paste and animal glue^6, 9^, respectively.

Here, we design a new scheme for efficiently removing stains including tideline, foxing, organic dyes and fungi from paper artworks by incorporating electrochemical reactions into the hydrogel-based cleaning process. Generally, electrochemical reactions have significant advantages over traditional chemical reactions. For instance, the catalyst/electrode is immobilized, thus reducing the requirement to separate the catalyst from the reaction mixture; the variable parameters in current and potential are readily controlled, hence easily controlling the desired reaction; the main reagent is the hydroxyl radical, which can efficiently degrade the organic stains but without a hazardous product. Meanwhile, the desired cleaning area can be regulated by using the miniaturized hydrogel electrode. Inspired by the following ideas, the electrode system, being composed of the PbO_2_ electrode as anode and reduced graphene oxide/polyacrylamide/montmorillonite conductive composite hydrogel pen (rGPM) as cathode, was employed for the cleaning of paper-based artifacts. (1) PbO_2_ is the most extensively used anode material for electrochemical oxidation of organic pollutants, because of its favorable electrical conductivity and high electro-catalytic activity in the formation of hydroxyl radical^16^. (2) The rGPM possesses excellent mechanical strength and conductibility. It has been reported that the incorporation of exfoliated sodium montmorillonite in polyacrylamide hydrogels could evidently enhance the strength of hydrogels as the strong hydrogen bonding between them^17^, and the introduction of reduced graphene oxide into the hydrogel could promote its electrical conductivity^18–20^. For hydrogels used in the electrochemical cleaning of paper artworks, both excellent mechanical strength and electrical conductivity are needed to avoid hydrogel residues on the paper and to transfer electrons on the electrode for electrochemical reactions.

**Figure 1:**
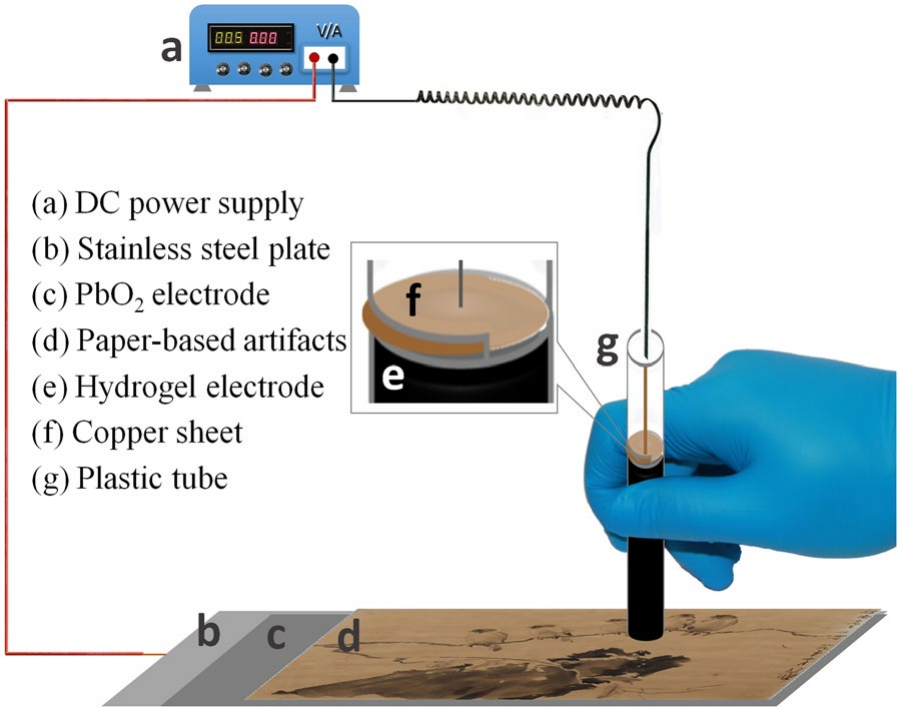
Schematic illustration of the HPEC method.

To our best knowledge, although the cleaning of water-sensitive cultural heritage artifacts by hydrogels^2–5^ and the degradation of contaminants by electrochemical reaction have been reported^21^, the hydrogel pen for electrochemical cleaning (HPEC) has never been presented. As illustrated in Fig. 1 for the HPEC, the PbO_2_ electrode was placed under the paper to be processed, and the rGPM was attached on the area expected to be cleaned. Namely, the paper with stains was sandwiched between the two electrodes. During cleaning, a consistent DC current was charged to the pair of electrodes to generate a given current density for the electrochemical cleaning. After a given time, the cleaning was finished and the rGPM electrode could be readily removed from the paper. To investigate the feasibility and adaptability of HPEC, we used the model materials such as new and artificially aged Chinese rice paper with stains including organic dyes, commercial drinks, tideline and mildew for the cleaning test. For reasons of security in cleaning of paper-based artifact, the effect of HPEC on the morphology and the chemical structure of the paper cellulose was assessed. Moreover, the reusability of HPEC was also evaluated. Based on the above investigations, HPEC was used to clean an actual paper-based artwork with stains including foxing, tideline and mildew, confirming the effectiveness of HPEC. The results showed that HPEC had an outstanding capacity for cleaning local stains formed by organic contaminants without an obvious influence on the cellulose and original mineral pigments. Additionally, the prepared PbO_2_ anode and rGPM cathode could be reused multiple times without loss of its efficiency. This work provides an alternative method for efficiently cleaning paper artworks.

## Results and Discussion

### Components and interactions in the reduced graphene oxide/polyacrylamide/montmorillonite composite hydrogel (rGPM)

**Figure 2:**
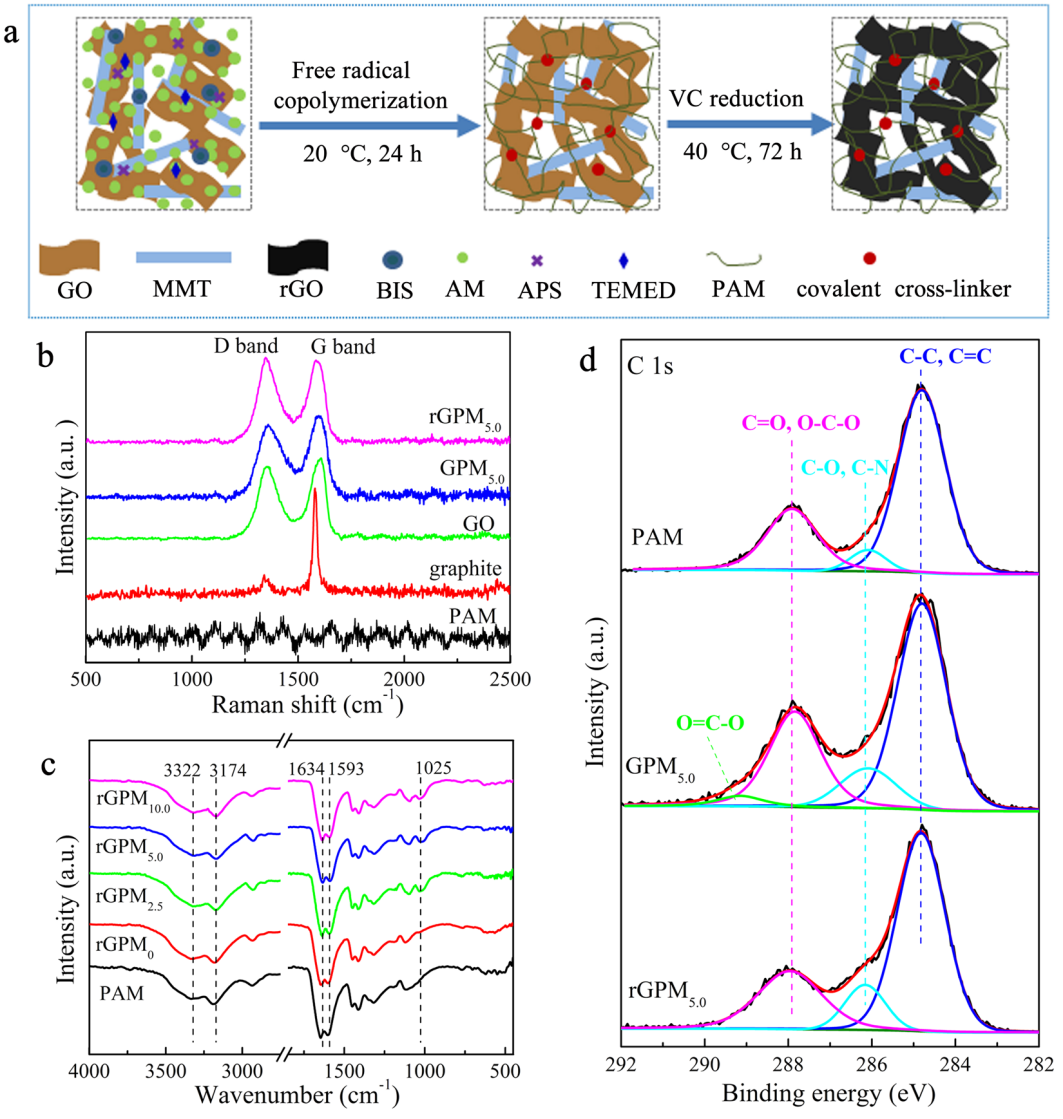
Schematic illustration of the preparation of rGPM_n_ hydrogels (**a**); Raman spectra of PAM, graphite, GO, GPM_5.0_ and rGPM_5.0_ (**b**); FT-IR spectra of PAM and rGPM with different content of MMT (**c**); C 1s XPS spectra of PAM, GPM_5.0_ and rGPM_5.0_ (**d**).

According to the process as illustrated in Fig. 2a for the fabrication of rGPM, the brown graphene oxide/polyacrylamide/montmorillonite composite hydrogel (GPM) was firstly synthesized by free radical polymerization of acrylamide (AM) and *N*,*N*′-methylenebisacrylamide (BIS) in the dispersion of graphene oxide (GO) and sodium montmorillonite (MMT). The prepared GPM possesses both chemical cross-linking and physical cross-linking. The chemical cross-linking created by BIS supports the stable hydrogel, and the physical cross-linking mainly based on the hydrogen bond interactions between polyacrylamide (PAM) chains and nanosheets of MMT and GO improves the mechanical strength of the hydrogel^17^. Then, the GO in GPM was reduced by L-ascorbic acid (VC) to form a black rGPM with enhanced electroconductivity^18^.

The graphene oxide (GO) was prepared according to the modified Hummers’ method^22^. The spectra of FT-IR (Supplementary Figure S1) and XPS (Supplementary Figure S2) indicated that the prepared GO has abundant oxygen-containing functional groups (such as (–COC–), (–OH) and (–COOH)) compared with the graphite. Furthermore, XRD patterns (Supplementary Figure S3) revealed an outstanding peak at 26.3° attributed to d-spacing of 0.34 nm for graphite and an evident peak at 9.8° assigned to d-spacing of 0.90 nm for GO, also indicating the presence of oxygen-containing functional groups on the GO nanosheets^33^. All these results suggest that GO has been successfully prepared.

It has been proven that MMT nanoplatelets enhance the mechanical strength of PAM hydrogels because of strong hydrogen bonds between amide side groups on PAM and units such as Si–OH and Si–O–Si on MMT^17^. Indeed, these interactions were also evidenced in the prepared GPM and rGPM. The diffraction peak at 7.2° ascribed to the basal spacing (*d*_001_, 1.2 nm) of MMT was shown in Supplementary Figure S3. However, any diffraction peak from 3 to 12° was not observed for GPM and rGPM, indicating that the lamellar structure of MMT was destroyed and dispersed uniformly in the composite hydrogels owing to the interactions between PAM chains and MMT^17^. Actually, these interactions were also confirmed by FT-IR spectra as shown in Fig. 2c. Peaks at 3336, 3190, 1649 and 1602 cm^−1^, severally attributed to the asymmetrical N–H stretching, symmetrical N–H stretching, amide-I (C=O stretching), and amide-II (N–H bending) of PAM^30, 31^, gradually shifted to 3322, 3174, 1634 and 1593 cm^−1^ with the increase of MMT in rGPM. In contrast, the band at 991 cm^−1^ originated from the Si–O–Si stretching of MMT^32^ (Supplementary Figure S1) was replaced by the peak at 1025 cm^−1^ in rGPM. These results from FT-IR spectra are in good agreement with the previous works^17, 33^, suggesting the strong interactions of hydrogen bonding between PAM and MMT. C 1 s XPS spectra of PAM, GPM_5.0_ and rGPM_5.0_ are given in Fig. 2d. For GPM_5.0_, the deconvolution of C 1 s peak presented four peaks at 284.8, 286.1, 287.9, and 289.2 eV, attributed to C–C/C=C, C–O/C–N, C=O/O–C–O, and O–C=O, respectively^34^. The relative amount of the bound carbons with oxygen increased from 32.9% for PAM to 44.5% for GPM_5.0_ due to the introduction of the rich oxygen-containing GO and MMT, decreased from 44.5% for GPM_5.0_ to 38.2% for rGPM_5.0_ owing to the reduction of GO^19^. Additionally, the peak at 289.2 eV attributed to O–C=O appeared for GPM_5.0_ and disappeared for rGPM_5.0_. These observations suggest that the reduction of GO in the hydrogel was proceeded by removing the oxygen-containing groups on GO.

Raman spectra for the involved materials are displayed in Fig. 2b. The presentation of both G and D band in the graphene-based samples indicates that graphene-based unit has been successful incorporated into the PAM matrix. Additionally, the ratio (*I*_D(1358 cm−1)_/*I*_G(1600 cm−1)_) was 0.896 for GPM_5.0_ and 1.304 for rGPM_5.0_, suggesting that the GO in GPM_5.0_ was partially reduced to the graphene in rGPM_5.0_^35^. The results shown in Supplementary Tables S1 and S2 indicated that the conductivity of rGPM_5.0_ was greater than that of GPM_5.0_, further confirming the conversion of GPM_5.0_ into rGPM_5.0_.

**Figure 3:**
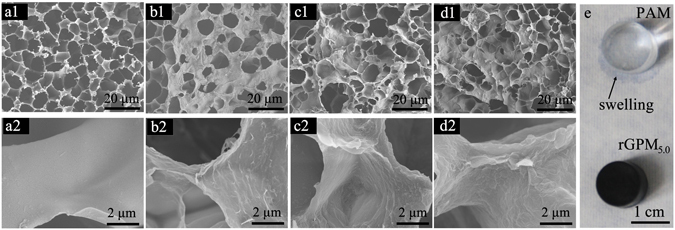
SEM images of PAM (**a1**,**a2**), rGPM_0_ (**b1**,**b2**), rGPM_2.5_ (**c1**,**c2**) and rGPM_5.0_ (**d1**,**d2**); the photos of PAM (top) and rGPM_5.0_ (bottom) placed on the paper for 6 min (**e**).

SEM images of the freeze-dried hydrogels are shown in Fig. 3. For the PAM hydrogel (Fig. 3a1), a typical porous structure with smooth pore wall was found. As shown in Fig. 3b1, the porous density of rGPM_0_ obviously decreased in comparison to PAM, and some flakes immersed in the pore wall, implying that graphene flakes were well-dispersed in the PAM hydrogel. As the addition of MMT into the hydrogel (Fig. 3c1–d1), the pore density of the hydrogel further decreased, and flakes on the pore wall became denser (Fig. 3c2–d2). These results suggest that the covalently crosslinked PAM chains are intertwined with MMT and graphene, which leads to a stiff gel to prevent the growth of ice crystals during the freezing step. Compared to PAM, rGPM_5.0_ with less and smaller interconnected pores to trap water showed a better water retention as it was attached on the paper (Fig. 3e).

### Mechanical performances of rGPM_n_

**Figure 4:**
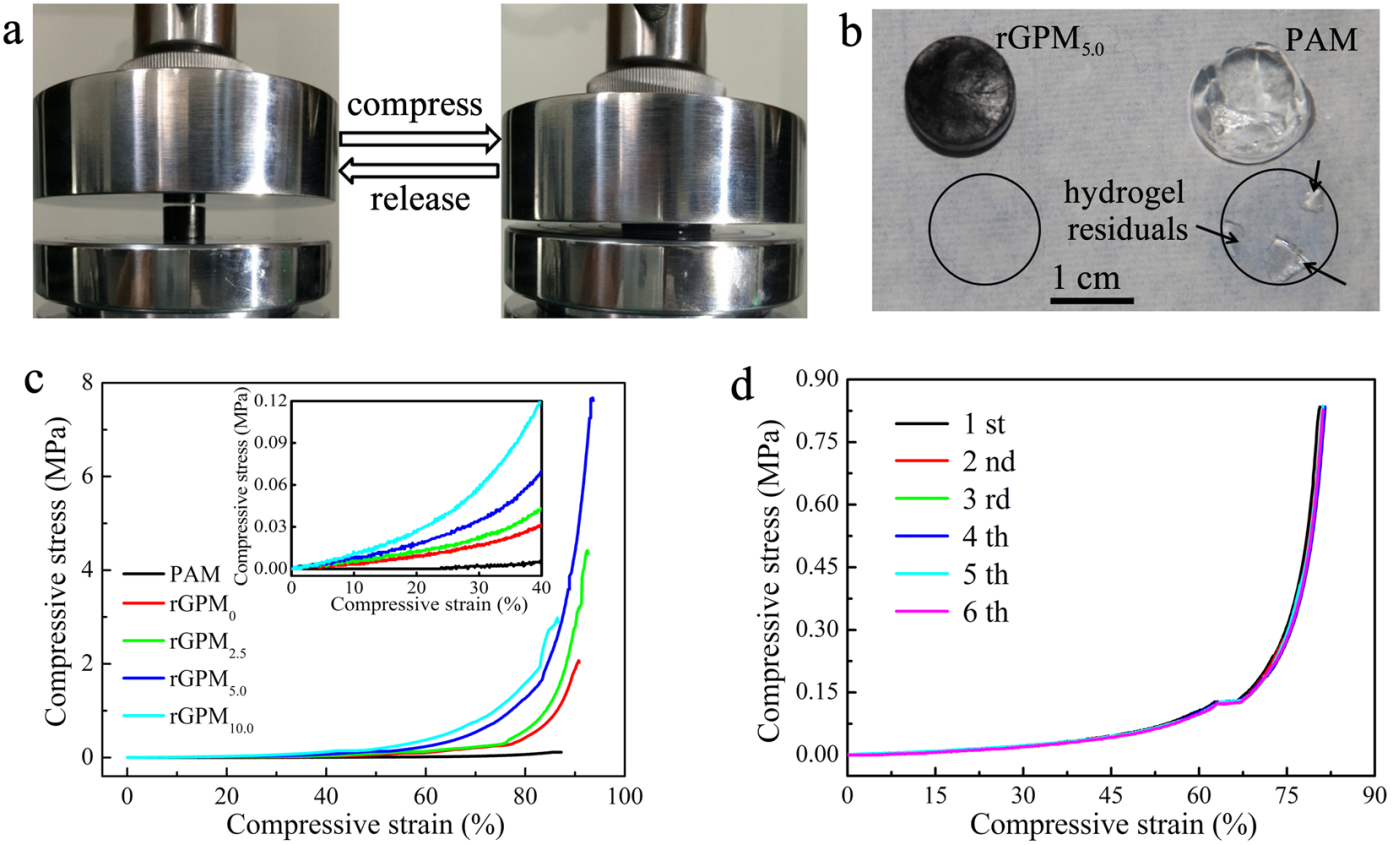
Photographs showing the rGPM_5.0_ hydrogel before and after compressing at *ε* = 85% (**a**). Photos demonstrating the residues of rGPM_5.0_ and PAM hydrogel after placed on a paper under a stress about 20 kPa for 10 min (**b**). Typical compressive stress–strain curves of PAM and rGPM (**c**). Stress–strain curves of rGPM_5.0_ hydrogel under consecutively repeated loading at *ε* = 80% (**d**).

For the hydrogel-based cleaning, the hydrogel with excellent mechanical property is necessary so that it can be readily peeled off from the paper without residues^4^. To show the excellent mechanical performance, Fig. 4a offered the photos of rGPM_5.0_ exhibiting recovery after compressing to 85% strain. In addition, rGPM_5.0_ could be peeled from a Chinese rice paper without any visible residuals after it was pressed at a stress of ~20 kPa on the paper for 10 min (Fig. 4b). In contrast, the PAM hydrogel was destroyed to leave some fragments on the paper. These observations indicate that rGPM_5.0_ possesses a highly resilient mechanical property. To confirm the mechanical performances of rGPM_n_, compressive stress–strain curves, dynamic stress sweeps and dynamic frequency sweeps were carried out. These results are shown in Fig. 4c and Supplementary Figure S4. The corresponding values of compressive modulus (*E*, kPa), compressive stress (*σ*, MPa), strain (*ε*, %), fracture energy density (*U*, kJ/m^3^) and equilibrium swelling degree (ESD, g/g) for rGPM_n_ and GPM_n_ are listed in Supplementary Tables S1 and S2. The critical stresses (*τ*_c_, Pa), plateau moduli (*G*, Pa), and effective network chain density (*N*_e_, mol/m^3^) for the hydrogels of PAM and rGPM_n_ were obtained by the calculation according to the literature^36^ and summarized in Supplementary Table S3. Based on these results, the following conclusions can be drawn. (1) MMT enhances the mechanical properties of the composite hydrogel when its amount is less than 5.0%. This conclusion is based on the changes of parameters (*σ*, *ε*, *τ*_c_, *E* and *U*) with the amount of MMT, and the corresponding explanations are given as follows. The *N*_e_ increases with the content of MMT, indicating that MMT as physical cross-linkers directly binds to the polymer network, and the multi-site interactions are confirmed by the fact that the determined elastic modulus (*E*) is markedly larger than that of the calculated modulus by Guth-Gold model (Supplementary Figure S5)^36, 37^. Evidently, a higher cross-link density originated from excessive MMT in the composite hydrogel leads to higher rigidity and lower toughness^26^. Therefore, a suitable amounts of MMT is inevitable to balance both rigidity and toughness of the composite hydrogel. (2) The increase of MMT results in the decrease of ESD, which is corresponding to the enhancement of crosslinking degree of hydrogel network^18, 26^.

Because of the removal of oxygen-containing groups from GO surface, the hydrogen bonding between PAM chains and rGO nanoplates could be decreased, which resulted in a smaller value of *ε*, *σ*, and *U* for rGPM_n_ in comparison to GPM_n_ (Supplementary Tables S1 and S2). In spite of this, as shown in Fig. 4d, the consecutive cyclic loading-unloading curves of rGPM_5.0_ at the strain range of 0–80% still demonstrate the excellent fatigue resistance and perfect reversibility, implying that rGPM_5.0_ can be repeatedly used as a hydrogel electrode without loss of its mechanical strength. Additionally, rGPM_5.0_ possesses an obvious conductivity (about 1.9 mS/cm), as shown in Supplementary Table S1, which is comparable with that of both pure graphene hydrogel (4.9 × 10^−3^ S/cm)^22^ and reduced GO-poly(N-isopropylacrylamide)-clay hydrogel (2.6–4.8 mS/cm)^18, 20^, indicating that rGPM_5.0_ can be used as electrode materials.

### Stain-removing performance of HPEC

**Figure 5:**
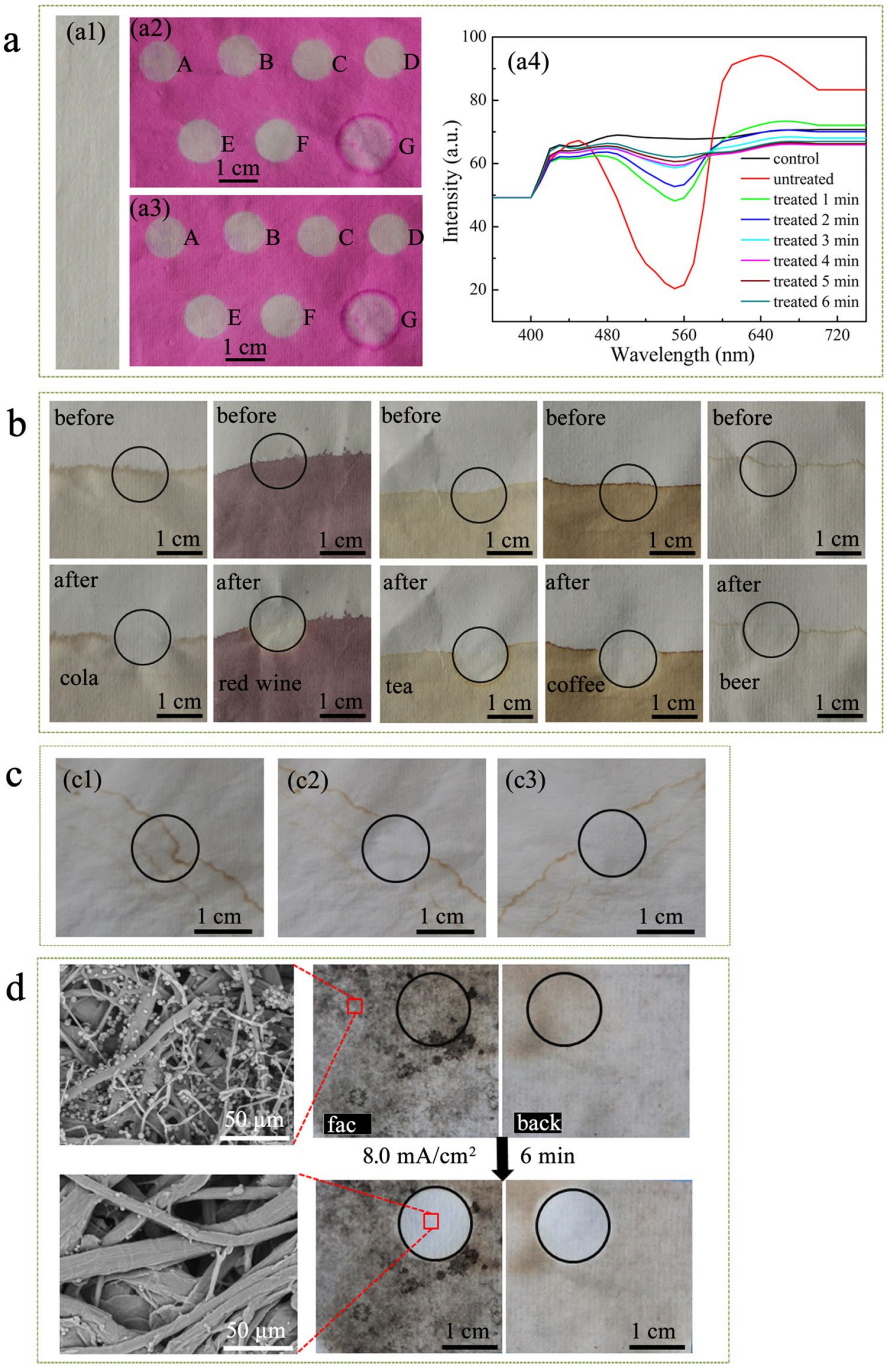
Performance of HPEC for cleaning safranine T from paper at 4 mA/cm^2^ (**a**). The control (a1). Face (a2) and back (a3) of the paper treated using HPEC for different time: A. 1 min, B. 2 min, C. 3 min, D. 4 min, E. 5 min, F. 6 min; G. treated for 2.0 h using the only PAM hydrogel. (a4) Normalized UV-vis reflectance spectra obtained from the areas corresponding to (a2). The efficiency of HPEC for cleaning stains of various drinks at 4 mA/cm^2^ within 5 min (**b**). The efficiency of HPEC for eliminating tideline (**c**). (c1) Before, (c2) face and (c3) back of the paper with tideline after treatment by using HPEC at 3.0 mA/cm^2^ for 3 min. The efficiency of HPEC for eliminating mildew (**d**).

Results of the safranine T-contaminated papers cleaned by HPEC at 4 mA/cm^2^ are shown in Fig. 5a. Figure 5a1,a2 and a3 are the photos of the undyed sample (**a1**), the surface contacted with rGPM_5.0_ electrode (**a2**) and the surface attached to the PbO_2_ electrode (**a3**) after cleaning treatment for different time, respectively. Obviously, the cleaning was only performed in the area contacted with the electrode, and synchronously occurred on both sides of the paper with the almost same effect. The UV-vis reflectance spectra of the corresponding areas as shown in Fig. 5a2 are presented in Fig. 5a4. These spectra of the treated area gradually tend to that of the control as the increase of cleaning duration. Using the control as reference, the colour difference (ΔE*) of the contaminated areas before and after cleaning are listed in Supplementary Table S4 together with the corresponding colorimetric data. The ΔE* of the cleaning area gradually decreased with the treatment time of HPEC. Generally, a ΔE* of 3.3 is the upper limit for human eyes to detect colour differences^38^. After a treatment of 6 min, the ΔE* of the area contacted with rGMP_5.0_ electrode was reduced from 54.03 (untreated area) to 3.56, suggesting that the color of the cleaning area is comparable to the control. As shown in Supplementary Figure S6, the excellent cleaning effect was also confirmed by the cleaning of the paper dyed with blue ink. However, as shown in Fig. 5a2(G) and a3(G), after a treatment with the PAM hydrogel for 2 h on the same dyed paper, the corresponding ΔE* values for the both sides of the paper were 27.41 and 33.65, respectively, and the safranine T diffused through water beyond the contacted area of PAM hydrogel.

The experimental results also indicated that the rGPM_5.0_ electrode could be repeatedly used for six times without any loss of efficacy (Supplementary Figure S7a) and the rGPM_5.0_ electrode could be prepared in different sizes to regulate the cleaning area (Supplementary Figure S7b). Additionally, it is worth noting that HPEC could only eliminate organic dye on the paper but without a visible effect on the mineral pigments (Supplementary Figure S7c). Generally, the colors used in traditional Chinese painting are mineral pigments. It can be deduced from these case studies that HPEC does not noticeably impact the traditional Chinese painting itself.

To evaluate the performance of HPEC for various stains, different stains eliminated by HPEC were conducted, and the results are also shown in Fig. 5. As shown in Fig. 5b, various drink stains on the paper could be erased by HPEC at 4 mA/cm^2^ within 5 min. The tideline stains originated from the cellulose degradation products not only detract the aesthetics of paper artworks but also accelerate the degradation of cellulose^24^. The excellent performance of HPEC in cleaning of tideline stains was also found (Fig. 5c). Evidently, the tideline stains on the both sides of paper are completely eliminated by using HPEC at 3.0 mA/cm^2^ within 3 min. However, although the PAM hydrogel attached on the paper for 3.0 h could slightly remove tideline stains, fulvous contaminants were diffused beyond the contacted area to generate new tideline stains (Supplementary Figure S8).

Molds and mildews not only deteriorate the aesthetics of paper artworks but also further decompose the paper cellulose^39^. To remove molds and mildews, various protocols including mechanical cleaning^40^, freeze-drying^41^, laser^42^, UV irradiation^43^ and biocleaning^44^, have been developed. However, these methods still have certain issues such as low cleaning efficiency and impacting on the paper itself. Interestingly, the mildew stains on both sides of the paper could be distinctly not only eliminated (Fig. 5d) but also inactivated (Supplementary Figure S9) by using HPEC at 8 mA/cm^2^ within 6 min.

**Figure 6:**
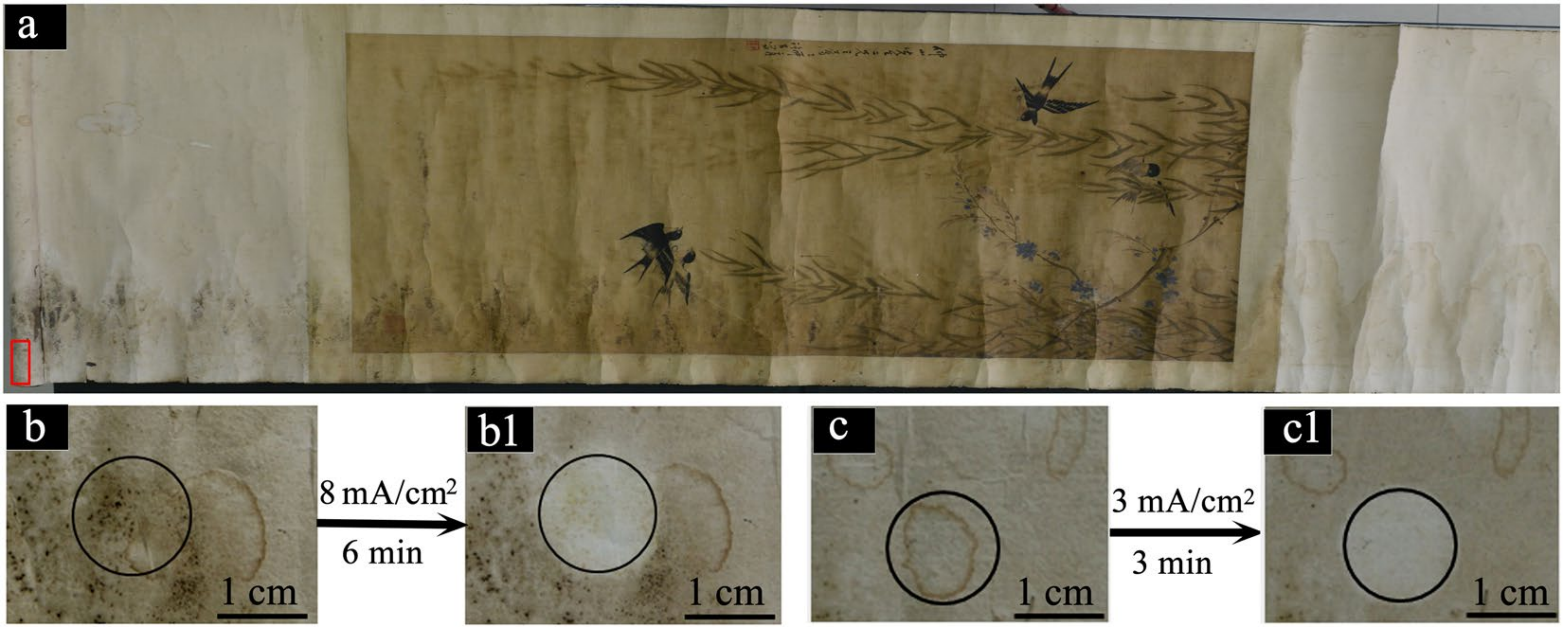
Performance of HPEC for eliminating various stains (mildew, foxing and tideline) on a real artwork. (**a**) A Chinese painting created by Xu Shi (Qing Dynasty, ~1850); (**b**) an original piece with mildew stains collected from the painting (red box region); (b1) the piece with mildew stains after treatment by using HPEC at 8.0 mA/cm^2^ within 6 min; (**c**) an original piece with foxing stains collected from the painting (red box region); (c1) the piece with foxing stains after treatment by using HPEC at 3.0 mA/cm^2^ within 3 min.

The excellent efficiency of the new method was also confirmed in practical applications. As a real example, results for cleaning various stains (mildew, tideline, foxing) on a Chinese painting (created by Xu Shi, Qing Dynasty, ~1850) by using HPEC are shown in Fig. 6. Obviously, the black mildew stains together with the yellowish foxing stains were almost completely eliminated at 8 mA/cm^2^ for 6 min (Fig. 6b1), and the tideline stains could be thoroughly removed at 3 mA/cm^2^ within 3 min (Fig. 6c1).

All above results indicate that HPEC is well adaptive and efficient to locally clean various stains on the paper artworks under mild conditions.

### Stain-removing mechanism of HPEC

**Figure 7:**
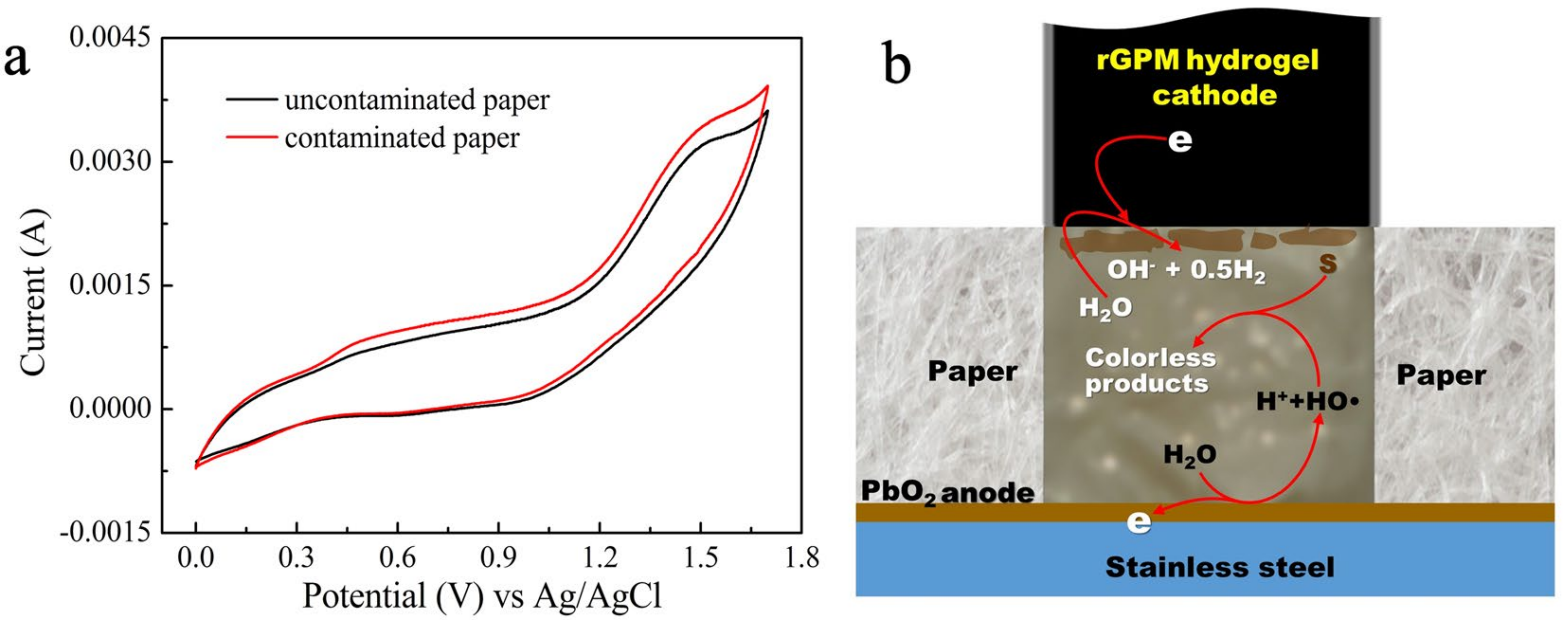
The typical cyclic voltammograms of HPEC process (**a**) and a proposed mechanism of the elimination of stains during HPEC process (**b**). A the safranine T undyed and dyed paper were respectively inserted between rGPM_5.0_ cathode and the PbO_2_ anode, and the scan rate was 50 mV/s.

Employing the prepared PbO_2_-based electrode as working electrode, rGPM_5.0_ as counter electrode and Ag/AgCl as reference electrode, the cyclic voltammetry curves recording for the papers with and without contamination of safranine T are shown in Fig. 7a. Similar to the cyclic voltammetry curves of PbO_2_-based electrode used in the degradation of organic pollutants^45^, any new anodic current peak was not found in the curve for the dye-contaminated paper in comparison to the uncontaminated one, indicating that safranine T is not oxidized directly on the surface of PbO_2_ anode but decomposed by hydroxyl radicals generated through the anodic discharge of water on the surface of PbO_2_ electrode^45^. The formation of hydroxyl radicals in this case was verified by the sodium terephthalate (ST) fluorescence probe method. As illustrated in Supplementary Figure S10, an obvious emission peak at 426 nm derived from ST oxidized by hydroxyl radicals was found^46^, indicating the formations of hydroxyl radicals during HPEC procedure.

Actually, hydroxyl radical is an powerful oxidant to degrade various organic compounds. During this process, organic compounds are broken down through a series of reactions including hydroxylation, decarboxylation, dehydration, demethylation and ring-opening reaction and even mineralization^47^. Based on our investigation and the conclusions in literatures^45, 47^, a stain-removing mechanism of HPEC as shown in Fig. 7b was proposed. The hydroxyl radicals generated on the surface of PbO_2_ anode diffuse into the soiled paper between the two electrodes and react with contaminants (S) to form some colorless products. For the local release of water from rGPM_5.0_ electrode, the degradation reactions occur only in the electrode attached area. It is worth noting that the pigments used for Chinese painting are usually derived from minerals. Mineral pigments are hardly oxidized in the high standard electrode potential of HO˙/H_2_O (2.80 V)^48^ and, therefore, HPEC can not change the mineral pigments.

### Effect of HPEC on the paper fibres

To assess the possible damage of the paper caused by cleaning processes, the effect of HPEC on the paper fibers was investigated.

**Figure 8:**
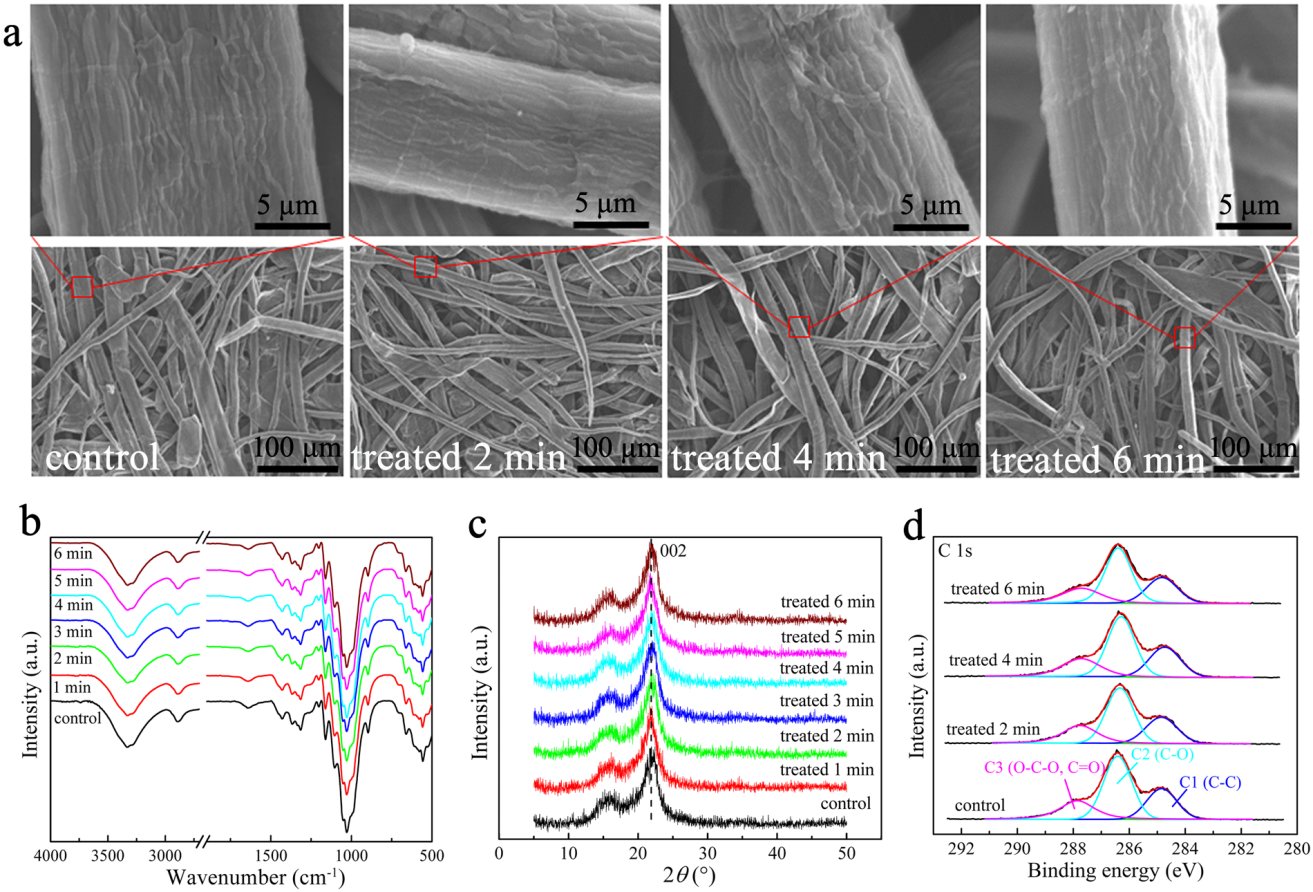
SEM images (**a**), FT-IR spectra (**b**), XRD patterns (**c**) and C 1 s XPS spectra (**d**) for the control paper and the paper treated by HPEC with different time.

Figure 8a displays the representative SEM images of the papers treated by HPEC at 4 mA/cm^2^ for different time. Clearly, for the papers treated with various time, the boundary of fiber bundle is almost perfect and coherent, which is closely similar to that of the untreated one. Additionally, the determining results shown in Supplementary Figure S11 indicated that HPEC scarcely alters the degree of polymerization (DP) of the paper cellulose.

FT-IR spectra for the papers with different treatments are presented in Fig. 8b. Compared with the FT-IR spectrum of the control, no new peaks appeared in FT-IR spectra of the papers treated with different durations, demonstrating that no new detectable functional groups generated during HPEC. The Total Crystallinity Indices (TCI, *I*_1372_/*I*_2903_)^49^ for all paper samples were calculated based on the ratio of intensity at 1372 cm^−1^ and at 2903 cm^−1^ and listed in Supplementary Table S5. TCI levels for the treated papers were approximately equal to that of the control, suggesting that HPEC dose not affect the crystallinity of the paper cellulose. This conclusion was further confirmed by XRD experiments. As shown in Fig. 8c, for all treated papers, the typical diffraction patters of native cellulose (or cellulose I) were found, and the peak at about 16.0° assigned to the (110) lattice plane in the triclinic structure as well as the peak at about 22.0° ascribed to the (200) lattice plane in the monoclinic structure appeared^49^, which are similar to that of the untreated paper. According to the methodology described in the literatures^50, 51^, Crystallinity Indices (CrI) and Crystallite Sizes (*L*_002_) for all samples were calculated and listed in Supplementary Table S5. Compared with the control, the CrI and *L*_002_ of the treated papers were almost unchanged. These results also support the conclusion that the fibers are hardly affected by the given HPEC process. The high-resolution C 1 s XPS spectra of the papers treated with different durations are given in Fig. 8d. For all papers, three peaks at about 284.8, 286.3 and 287.8 eV respectively attributed to (C–C), (C–O) and (O–C–O and/or C=O) were found^52^, and these peaks were almost the same as that of the control, revealing that the proposed PHEC treatment does not cause a significant oxidation of the paper fibres.

In fact, hydroxyl radicals can degrade paper through autocatalytic reaction on the long time scale (years)^10^. In the current work, however, the treatment is very mild, and the treated duration is a few minutes. Based on our experimental results and reports in literature^48^, it is reasonable to believe that the influence of HPEC on the paper cellulose is slight.

### Effect of HPEC on the mechanical performance of paper

The stress-strain curves as well as the corresponding values of ultimate tensile strength (*σ*_t_), and breaking strain (*ε*_t_) for the papers with different treatment are shown in Supplementary Figure S12 and Supplementary Table S6, respectively. The *σ*_t_ of the HPEC treated paper mildly decreased with the increase of treatment time, but the corresponding *ε*_t_ increased slightly, indicating that PHEC treatment does not cause a significant decay in the mechanical performance of the paper. Meanwhile, both stress-strain curves of the papers treated for 6 min by HPEC and by the only rGPM_5.0_ were almost overlapped, suggesting the same effect of the two procedures on the mechanical strength of the paper. However, the paper showed a significant decrease for *σ*_t_ and an evident increase for *ε*_t_ in comparison to the control one when it was dipped in water for 6 min. In fact, here the slight change of the mechanical performance for the paper treated by HPEC may be attributed to the difference in drying history. It has been reported that a wetting-drying cycle, more or less, makes fibers overlap less and bent, resulting in a dramatic decay in *σ*_t_ and a significant increase in *ε*_t_^1, 5^. In the current work, the released water (i. e. the weight increment of the paper sheet after the treatment, Δ_W_) from the rGPM_5.0_ electrode slightly increased with the increase of HPEC treatment time (Supplementary Table S6), resulting in a mild increase in change of the fiber texture. This morphology change could be found in Fig. 8a. In any case, the effect of HPEC treatment on the mechanical performance is weak, and this effect mainly originates from water rather than the electrochemical process itself.

## Conclusion

In summary, by using the graphene based composite hydrogel and PbO_2_-based material as electrode, we develop a simple and efficient system for electrochemically cleaning paper-based artworks. The proposed method has significant advantages over traditional methods. (1) The variables in current and potential are readily controlled, hence easily controlling the desired reaction for treating different stains. (2) The main reagent is hydroxyl radical, which degrades the organic stains, even the intractable mildew stains, within several minutes. For a short-term treatment, the influence of HPEC on the paper cellulose is very slight. (3) The miniaturized hydrogel electrode can tailor the electrochemical reaction not only in the desired area but also the expected reaction. (4) Owing to the special cleaning mode and the outstanding mechanical performances, the composite hydrogel electrode can be easily peeled off from the surface of paper without any residues after cleaning, and can be repeatedly used for several times without loss of efficiency. From aforementioned points of view, we constructed a electrochemistry method with peculiar characteristics for cleaning paper-based artifacts. This process can remove the strains including organic dyes, drinks, tideline, foxing and mildews from paper-based artworks in a controllable area. However, for safety, we suggest that this method should be first applied in a mimic to evaluate the influence of HPEC on the artwork itself before application.

## Methods

### Materials

Acrylamide, and *N*,*N*′-methylenebisacrylamide were purchased from Sigma-Aldrich and used as received. Graphite powder (100 meshes, 99.99%), ammonium persulfate (APS), *N*,*N*,*N*′,*N*′-tetramethylethylenediamine (TEMED), L-ascorbic acid (VC), and other reagents were of analytical grade and used as received from Sinopharm Chemical Reagent Co. Ltd. Sodium montmorillonite (MMT) was supplied by Zhejiang SAND Technology Co. Ltd. and used after washing and freeze-drying. Ultrapure water (18.2 MΩ at 25 °C) was used throughout the experiments. 316 stainless steel plates with a size of 150 × 35 × 2 mm were obtained commercially. Chinese rice papers (Xuanzhi in Chinese), widely used in Chinese traditional painting and calligraphy, were purchased from Chongxing Xuanzhi Factory and used as model paper samples. The used Xuanzhi with a density of 45.3 g/m^2^ was made of sandalwood pulp (>75%) with trace amount of Chinese alpine rush.

### Synthesis of hydrogel-based electrode

Graphene oxide (GO) was firstly prepared from graphite powder according to the literature^22^, and then an aqueous dispersion of GO and exfoliated MMT was prepared by mixing 10.0 mL GO (8.0 mg/mL) and a certain amount of MMT under ultrasound for 2.0 h. Subsequently, 2.0 g AM and 4.0 mg BIS were added into the dispersion and stirred in ice-water bath for 3.0 h. After purging with nitrogen gas for 30 min, an aqueous solution of APS (100 μL, 0.1 mol/L) and TEMED (10 μL) were successively added into the dispersion. The resultant mixture was rapidly injected into a sealed plastic tube with different diameters, and the columnar GO-poly(acrylamide)-MMT nanocomposite hydrogel (GPM) was formed at 20 °C for 24 h.

GPM hydrogels were immersed in the aqueous solution of VC (20 mg/mL) at 40 °C for 3 days to form reduced GO-poly(acrylamide)-MMT nanocomposite hydrogels (rGPM)^18^, and then rGPM were washed with water to remove excessive VC. To explore the effect of MMT on the mechanical strength of rGPM, rGPM composite hydrogels with different MMT content were synthesized by the analogous procedure and they were designated as rGPM_n_ (here n refers to the weight percentage of MMT to AM). For comparison, poly(acrylamide) hydrogel (PAM) was also prepared in the absence of GO and MMT.

### Fabrication of PbO_2_ electrode

The PbO_2_ electrode was prepared according to the literature procedure^23^.

### Preparation of papers with simulated stains

To assess the HPEC performance, Chinese rice papers with various simulated contaminants including organic dyes, commercial drinks (coffee, tea, cola, and red wine), tideline, and mildew were prepared using the following processes.

Simulated papers contaminated with different organic dyes were prepared by soaking Chinese rice papers in safranine T solution (1.0 wt%) or in blue ink for 1.0 h. After removing from the solution and drying in air, the desired papers were gained. Analogously, commercial drinks (coffee, tea, cola, and wine) were brushed on Chinese rice papers, and then the corresponding various stains on the papers were produced after the evaporation of water.

Molds were carefully collected from a book of our library and incubated by potato dextrose agar (PDA) at 28 °C for 10 days. The colonies were separated and dispersed in water to form a mold suspension. Papers after immersion in the suspension were pasted on the fresh PDA and cultivated for 8 days in a incubator with a relative humidity (RH) of 95% at 28 °C. Then, the papers contaminated with mildew were obtained after they were peeled from the surface of PDA and dried at room temperature.

Chinese rice papers were first treated by moist heat aging at 80 °C and 90% RH for 7 days. In this process, yellowish or brownish products of cellulose degradation in the papers were formed. A few drops of water were dropped onto the aged paper, and then the paper with tideline stains were obtained after drying at room temperature because of the diffusion of cellulose degradation products at the wet/dry interface^24^.

### Actual paper-based cultural relic with stains

A Chinese painting created by Xu Shi (Qing Dynasty, ~1850) exists various stains including mildew, foxing and tidelines. As a cleaning example of actual paper-based cultural relic, this painting was selected to evaluate the performance of HPEC.

### Characterizations

The relevant properties of the composite hydrogels, the cleaning performance of HPEC and the effect of HPEC on the paper, were characterized.

### The swelling behavior of composite hydrogels

The morphologies of freeze-dried hydrogels were observed by using a Hitachi SU-8020 field emission scanning electron microscopy. The equilibrium swelling degree (ESD, g/g) of the swollen hydrogel was calculated from the equation (1).

1 $${\rm{ESD}}=({W}_{{\rm{e}}}-{W}_{{\rm{d}}})/{W}_{{\rm{d}}},$$

where *W*_e_ and *W*_d_ represent the weights of the swollen hydrogel and the corresponding dried hydrogel, respectively.

### The composition of composite hydrogels

Raman spectra of the involved materials were obtained by using a Renishaw inVia Reflex microspectrometer with an excitation wavelength of 532 nm. Fourier transform infrared (FT-IR) spectroscopy was recorded in the range from 500 to 4000 cm^−1^ using a Perkin Elmer FT-IR spectrophotometer with attenuated total reflectance (ATR) mode. X-ray photoelectron spectroscopy (XPS, Axis Ultra Kratos Analytical Ltd.) of the relevant composite hydrogels was performed using Al Kα radiation (1486.6 eV) as the excitation source. X-ray diffraction (XRD) experiments were carried out on a Rigaku D/Max-2500 X-ray diffractometer with Cu K*α* X-ray beam at 40 kV and 30 mA.

### The mechanical performances of composite hydrogels

Mechanical performances of the swollen hydrogels were measured with a universal mechanical testing apparatus (QT-1136, Dongguan Qualitest Instrument Co., Ltd.) in compression mode. A cylindrical sample (~15 mm in diameter and ~10 mm in height) was compressed at room temperature and a rate of 10 mm/min with a 1000 N load cell. The compressive modulus (*E*, kPa), compressive stress (*σ*, MPa), strain (*ε*, %) and fracture energy density (*U*, kJ/m^3^) were calculated from the stress–strain curves according to the literatures^25, 26^. The fatigue resistance of the swollen hydrogel was investigated by consecutive 6 cycles of compressive loading–unloading test at the strain range of 0–80%. Oscillatory shear measurements for the relevant hydrogels were performed on a TA Instruments AR-G2 rheometer equipped with parallel plate geometry (20 mm diameter) at 25 °C. Dynamic stress sweep experiments were carried out at the frequency of 1 Hz. Dynamic frequency sweep experiments were performed over the frequency range from 0.1 to 100 rad/s under a constant stress of 10 Pa.

### The electrochemical behavior of HPEC

The electrical conductivity of the composite hydrogels was determined by using a four-probe technique on a SX1934 digital multimeter (Suzhou Telecommunication Factory, China). Cyclic voltammograms were recorded in a three-electrode system using a CHI 760D electrochemical workstation (CH Instruments) at room temperature and a scan rate of 50 mV/s. The PbO_2_-based electrode, rGPM_5.0_, and Ag/AgCl were performed as working electrode, counter electrode, and reference electrode, respectively.

### The cleaning performance

The colorimetric coordinates (L*, a*, b*) of the papers were obtained by using a spectrophotometer (VS450, X-Rite) in the range from 360 to 750 nm. The degree of cleaning was evaluated through the color difference (ΔE*) that was calculated by the equation (2)^3^.

2 $${\rm{\Delta }}{E}^{\ast }={[{({\rm{\Delta }}{L}^{\ast })}^{2}+{({\rm{\Delta }}{a}^{\ast })}^{2}+{({\rm{\Delta }}{b}^{\ast })}^{2}]}^{0.5}$$

where ΔL*, Δa*, and Δb* are respectively differences between the measured Lab values of the non-soiled and the soiled or the cleaned sample.

### The microstructure and composition of cleaned papers

To explore the effect of HPEC on the paper, the morphology, composition as well as mechanical performances of the papers before and after HPEC treatment were measured. The morphology was observed on a scanning electron microscope (Quanta 200, FEI), and the composition was characterized by a Rigaku D/Max-2500 X-ray diffractometer, a Perkin Elmer FT-IR spectrophotometer with ATR mode, and a Kratos Axis Ultra X-ray photoelectron spectrometer. The degree of polymerization of paper cellulose was estimated by the determination of intrinsic viscosity as described in the literature^27^. Tensile tests were performed on a universal testing machine equipped with a 100 N load cell at a deformation rate of 20 mm/min. Before testing, all samples were dried in air for 24 h and then stored for at least 48 h in a desiccator with a RH of 58% to achieve a complete equilibrium. For comparison, the paper samples treated by the only rGPM_5.0_ hydrogel for 6 min and dipped in water for 6 min were also used for the tensile test.

### Data availability

All data generated or analyzed during this study are included in this published article (and its Supplementary Information file).

## Electronic supplementary material

Supplementary Information
