# Supplementary material for: Electrochemical removal of stains from paper cultural relics based on the electrode system of conductive composite hydrogel and PbO2
Source: Sci Rep. 2017 Aug 18;7:8865. doi: 10.1038/s41598-017-08907-w (PMC5562847; doi:10.1038/s41598-017-08907-w)
Supplement: Supplementary file 1 — Supplementary Information [file 41598_2017_8907_MOESM1_ESM.pdf]

Supplementary Information:

Electrochemical removal of stains from paper cultural relics based on the electrode system of conductive composite hydrogel and PbO_2

Xingtang Liang[1,2], Lizhen Zheng[1], Shirong Li[1], Xiaoyu Fan[1], Shukun Shen[1], and Daodao Hu [1,]*

[1]Engineering Research Center of Historical and Cultural Heritage Protection, Ministry of Education, School of Materials Science and Engineering, Shaanxi Normal University, Xi'an 710062, China

[2]School of Petroleum and Chemical Engineering, Qinzhou University, Qinzhou 535000, China

***Corresponding author**: daodaohu@snnu.edu.cn (D.H.).

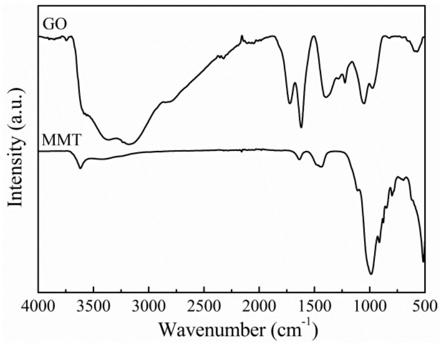

Figure S1 FT-IR spectra of GO and MMT. The GO presents peaks at 3188, 1719, 1618, 1405, and 1047 cm^{-1}, respectively, which correspond to hydroxyl stretching, C=O carbonyl stretching, aromatic C=C stretching, O–H deformation, and C–O stretching.

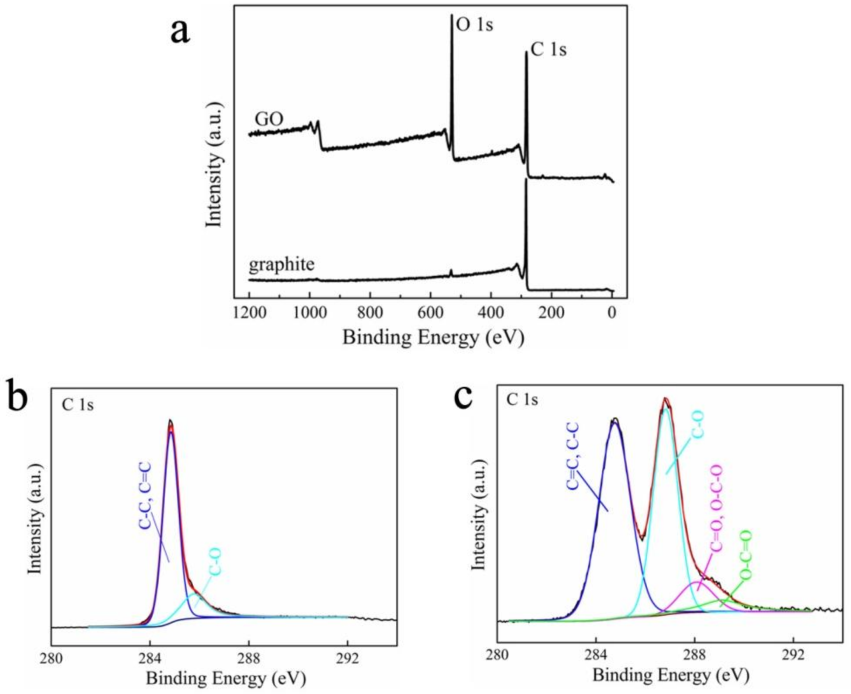

Figure S2 XPS spectra of GO and graphite (a); C 1s XPS spectra of graphite (b) and GO (c).

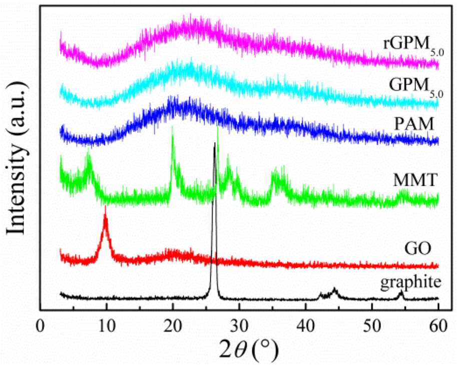

Figure S3 XRD patterns of graphite, GO, MMT, PAM, $GPM_{5.0}$ and $rGPM_{5.0}$.

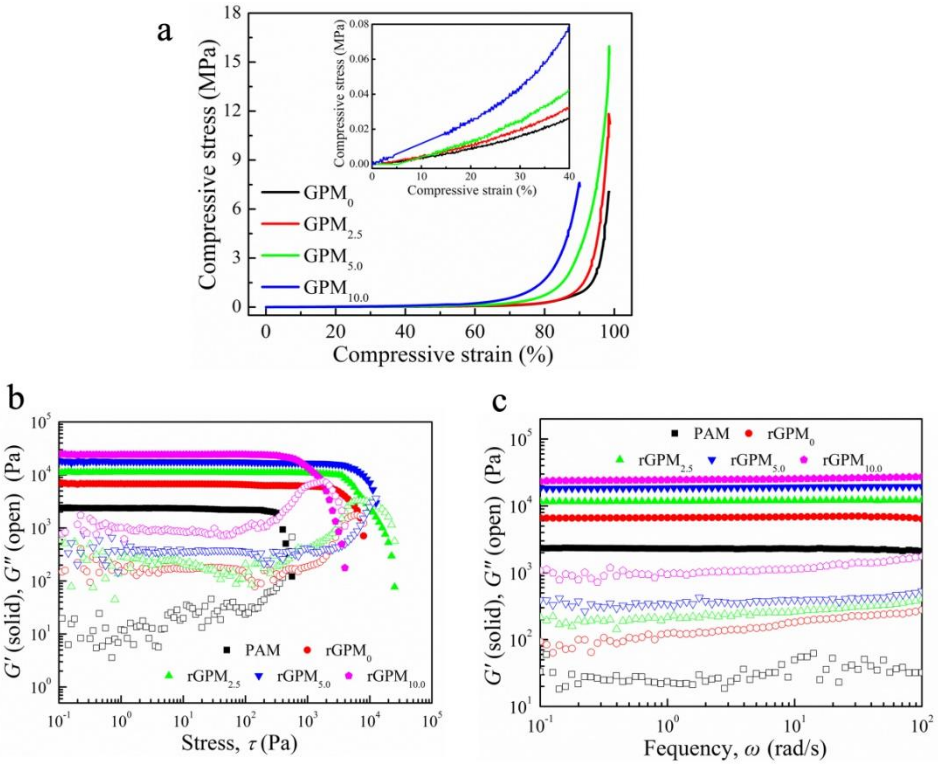

Figure S4 Typical compressive stress–strain curves of GPM hydrogels with different amount of MMT (a); Dynamic stress sweep (b) and dynamic frequency sweep (c) of PAM and rGPM hydrogels with different amount of MMT.

Table S1 Compressive properties, ESD, and conductivity of the PAM and rGPM hydrogels with different amount of MMT (mean ± standard deviation, n = 3).

sample code	GO[a] (mL)	MMT (g)	E (kPa)	ε (%)	σ (MPa)	U (kJ/m^3)	ESD (g/g)	conductivity (mS/cm)
PAM	10.0[b]	0	15.2 ± 3.2	86.2 ± 5.1	0.1 ± 0.02	12.5 ± 2.2	19.7 ± 0.7	not conductive
$rGPM_0$	10.0	0	80.9 ± 8.9	90.9 ± 2.3	2.0 ± 0.45	169.8 ± 15.9	15.6 ± 0.2	0.92 ± 0.29
$rGPM_{2.5}$	10.0	0.05	97.5 ± 20.6	92.5 ± 0.9	4.4 ± 0.89	302.3 ± 25.1	13.2 ± 0.4	1.16 ± 0.42
$rGPM_{5.0}$	10.0	0.10	173.2 ± 14.1	93.6 ± 0.7	7.6 ± 0.73	616.8 ± 21.4	12.5 ± 0.1	1.90 ± 0.21
$rGPM_{10.0}$	10.0	0.20	310.3 ± 18.8	86.6 ± 2.6	2.9 ± 1.25	371.5 ± 26.3	11.8 ± 0.3	2.14 ± 0.37

[a]: The concentration of GO is 8 mg/mL.

[b]: 10 mL water has be used.

Table S2 Compressive properties, ESD, and conductivity of GPM hydrogels with different amount of MMT (mean ± standard deviation, n = 3).

sample code	E (kPa)	ε (%)	σ (MPa)	U (kJ/m^3)	ESD (g/g)	conductivity (mS/cm)
GPM_0	66.5 ± 13.1	98.4 ± 1.1	7.0 ± 0.7	298.9 ± 18.5	13.8 ± 0.5	0.13 ± 0.08
$GPM_{2.5}$	90.1 ± 16.2	98.3 ± 1.6	11.8 ± 0.4	493.3 ± 29.7	12.1 ± 0.2	0.18 ± 0.12
$GPM_{5.0}$	127.5 ± 21.3	98.5 ± 0.9	16.0 ± 0.6	984.9 ± 42.8	11.6 ± 0.3	0.21 ± 0.16
$GPM_{10.0}$	190.6 ± 19.8	90.1 ± 1.5	7.6 ± 1.2	564.6 ± 35.1	10.7 ± 0.2	0.28 ± 0.09

Table S3 τ_c , G, and N_e for the PAM and rGPM hydrogels with different amount of MMT.

sample code	τ_c (Pa)	G (Pa)	N_e (mol/m^3)
PAM	320	2290	0.94
$rGPM_0$	2010	6810	2.80
$rGPM_{2.5}$	3210	12030	4.94
$rGPM_{5.0}$	3650	19080	7.83
$rGPM_{10.0}$	630	25510	10.5

Reduction decreases oxygen-containing groups of GO in the hydrogel and weakens the hydrogen bonds between PAM chains and GO to reduce the physical cross-linkers. Therefore, the compressive properties of $rGPM_n$ hydrogels including σ, ε, and U lose along with the increase of EDS in comparison to the corresponding GPM_n hydrogels. However, because rGO has more perfectly conjugated carbon atoms to boost its Young's modulus in comparison to GO,[1,2] the E of $rGPM_n$, signifying the rigidity of hydrogel, is higher than that of the corresponding GPM_n.

In stress-sweep dynamic rheology experiments (**Supplementary Figure S4b**), the τ_c of $rGPM_n$ increases as the MMT amount when it is less than 5%, while reduces as the MMT amount is 10%, which is consistent with the σ obtained from compression experiments. In frequency-sweep dynamic rheology experiments (**Supplementary Figure S4c**), for all hydrogels, the G' (storage modulus) considerably exceeds the G'' (loss modulus) across the overall frequency window and the G' is nearly independent from the frequency, confirming the gel nature.[3] The G', characterizing the rigidity of hydrogel, increases as the MMT content, which agrees well with the previous analysis of E based on the compressive experiments, implying the reinforcement of MMT. The effective network chain density (N_e, mol/m^3) consists of the physical entanglements and covalent cross-linkers, is estimated by the equation,[4] $N_e = G / R\,T$, where, G is the plateau modulus and derived from the curve of G' vs. ω, R is the gas constant and T is absolute temperature. The calculated N_e increases with the increase of MMT content, suggesting that MMT nanoplates directly bond to the polymer network and function as physical cross-linkers in the hydrogel.

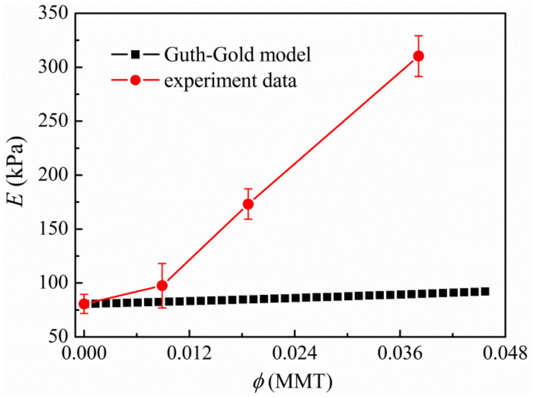

Figure S5 The experimentally compressive modulus and the predicted modulus derived from Guth-Gold model for rGPM hydrogels with different amount of MMT. The Guth-Gold model is described as $E = E_0\ (1 + 2.5\ \phi + 14.1\ \phi^2\ )$, where, E and E_0 are the predicted elastic moduli of MMT-loaded and unloaded hydrogels, respectively, ϕ is the MMT volume fraction.[5,6] This classic model offers a elastic modulus prediction for the rubbery matrix loaded with weakly interacting fillers. In this work, the experimental E for the MMT-loaded hydrogels are markedly greater than that of predicted modulus, indicating the strong hydrogen bonding interactions between PAM chains and MMT.

Table S4 Colorimetric values of L^*, a^*, b^*, and ΔE^* for the undyed original paper, safranine T dyed paper and the area of safranine T dyed paper after treatment by HPEC for different time[a].

sample code	L^*	a^*	b^*	ΔE^*
reference	91.75 ± 0.62	-0.68 ± 0.09	1.9 ± 0.12	–
untreated	73.36 ± 1.18	45.87 ± 0.84	-18.46 ± 0.25	54.03
treated 1 min	86.82 ± 1.45	11.26 ± 0.56	-4.06 ± 0.36	14.23
treated 2 min	88.96 ± 1.03	7.55 ± 0.71	-2.61 ± 0.16	9.79
treated 3 min	91.08 ±0.95	3.18 ± 0.12	-1.44 ± 0.11	5.14
treated 4 min	93.73 ± 0.83	1.29 ± 0.18	-1.41 ± 0.17	4.33
treated 5 min	91.96 ± 1.01	1.77 ± 0.23	-1.61 ± 0.24	4.31
treated 6 min	92.12 ± 0.80	0.72 ± 0.19	-1.35 ± 0.05	3.56

[a]: The presented colorimetric data (corresponding to **Fig. 5a2**) are the average value of 5 measurements.

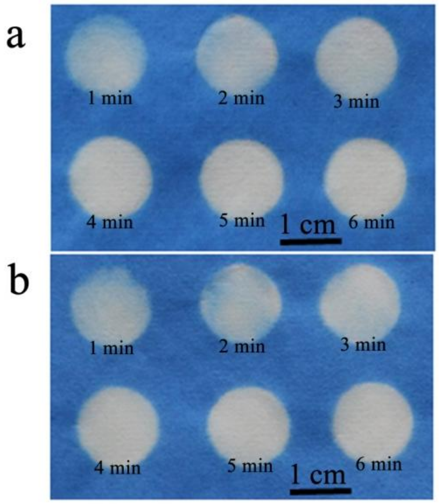

Figure S6. Photos for blue ink dyed paper cleaned by using HPEC at 4 mA/cm^2 for different time. The face (A) and back (B) of the paper treated with different time: a. 1 min, b. 2 min, c. 3 min, d. 4 min, e. 5 mint, f. 6 min.

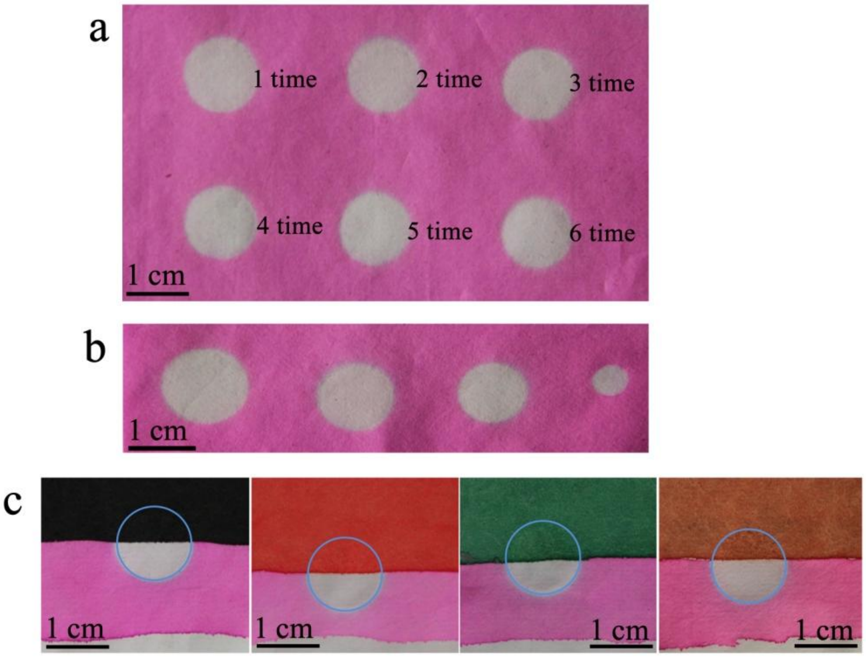

Figure S7. (a) Repeated performance of $rGPM_{5.0}$ hydrogel electrode for removal of safranine T in six cycles at 4 mA/cm^2 and 5 min. (b) Removal of safranine T from paper by HPEC with different sizes of $rGPM_{5.0}$ hydrogel electrodes under 4 mA/cm^2 and 5 min. (c) Photos illustrating the cleaning selectivity of HPEC (in every insert, inorganic pigment area (up) and organic dye area (bottom)). The mineral pigments are carbon black cadmium red, cobalt green, and ochre from left to right.

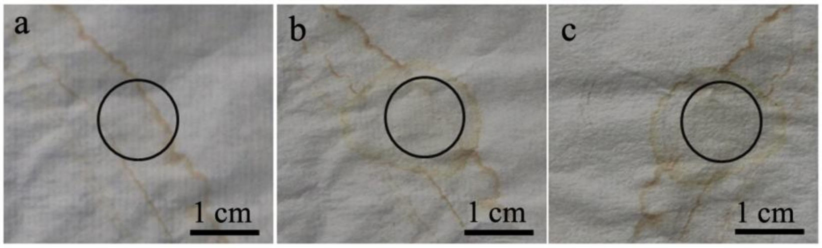

Figure S8. Photos indicating the efficiency of PAM for eliminating tideline. (a) Before, (b) the face and (c) back of the paper with tideline after treatment by only using PAM hydrogel for 3.0 h.

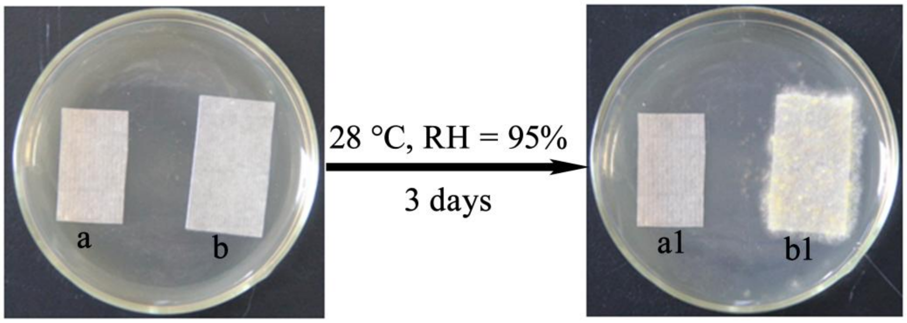

Figure S9. Photos showing the growth test of the spores on the papers with different treatments. (a) the mildew stained paper treated by using HPEC at 8.0 mA/cm^2 for 6 min; (b) the paper after dipping in the mold suspension; (a1) and (b1) severally corresponding to (a) and (b) after cultivation by PDA under a relative humidity of 95% and 28 °C for 3 days. The molds in the paper dipped in the mold suspension developed abundantly, while the remaining mold spores in the HPEC treated paper (**Fig. 5d**) did not grow, indicating that the molds in the treated paper were killed by HPEC.

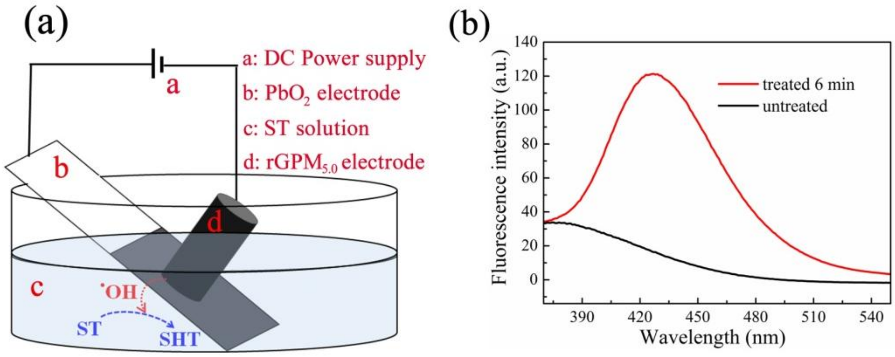

Figure S10. Schematic illustration of the detection of hydroxyl radicals during HPEC process by the sodium terephthalate (ST) fluorescence probe method. (a) The device for hydroxyl radicals formed in HPEC process to react with sodium terephthalate. (b) Fluorescence spectra of sodium terephthalate solution before and after HPEC treatment at 4 mA/cm^2 for 6 min. In this method, sodium terephthalate (a non-fluorescent compound) readily reacts with hydroxyl radicals to form a strong fluorescence product, sodium 2-hydroxyterephthalate (SHT), whose $\lambda_{excitation}$ and $\lambda_{emission}$ are about 316 nm and 426 nm, respectively.[7] Compared to the ST solution (5.0×10^{-4} mol/L), the fluorescence spectrum of the electrochemically treated ST solution exhibits an obvious peak at 426 nm excited by 316 nm light (**Supplementary Figure S10b**). This result confirms the formations of hydroxyl radicals during the HPEC process. In fact, hydroxyl radicals can efficiently inactivate the fungi by breaking the strands and modifying the nucleic base of the DNA of fungi.[8]

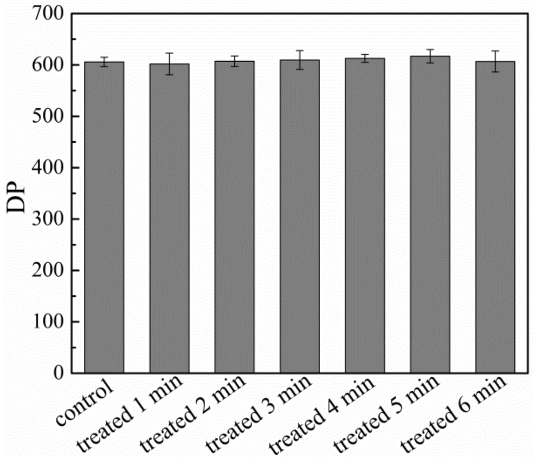

Figure S11. DP of the paper cellulose as a function of treated time at 4 mA/cm^2 (n = 3).

Table S5 Values of TCI, CrI, and L_{002} for the control paper and the papers treated by HPEC with different time. (mean ± standard deviation, n = 3).

paper sample	TCI	CrI (%)	L_{002} (nm)
control	0.517 ± 0.011	75.85 ± 0.58	3.51 ± 0.21
treated 1 min	0.512 ± 0.019	76.02 ± 0.32	3.54 ± 0.13
treated 2 min	0.506 ± 0.021	75.56 ± 0.47	3.50 ± 0.25
treated 3 min	0.509 ± 0.012	76.07 ± 0.89	3.76 ± 0.34
treated 4 min	0.506 ± 0.028	77.21 ± 0.75	3.53 ± 0.12
treated 5 min	0.510 ± 0.009	77.45 ± 1.01	3.64 ± 0.17
treated 6 min	0.524 ± 0.015	75.43 ± 0.64	3.68 ± 0.29

The crystallinity Indices (CrI) and the crystallite sizes (L_{002}) for the paper fibres were calculated according to equations of CrI = 100 (I_{002} – I_{am}) / I_{002} and $L_{002} = k\,\lambda / \beta \cos\theta$,[9] respectively, in which, I_{002} is the intensity of plane 002, I_{am} signifies the diffraction intensity of amorphous region at 18.0 º, k is the Scherrer constant (0.94), λ is the wavelength of X-ray (0.154 nm), β represents the full-width at half-maximum (002) peak and θ is the diffraction angle of (002) plane.

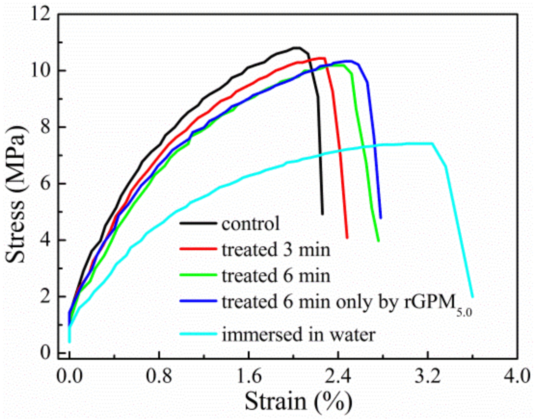

Figure S12. Typical tensile stress-strain curves for different papers, including the control paper; the papers treated by HPEC severally for 3 min and 6 min; the paper treated by the only $rGPM_{5.0}$ for 6 min; the paper immersed in water for 6 min. Before test, all samples were dried in air and completely equilibrated by the RH of 58% at room temperature.

Table S6 Values of σ_t, ε_t and weight increment (Δ_W)[a] for the paper samples with different treatment. (mean ± standard deviation, n = 3).

paper sample	σ_t (MPa)	ε_t (%)	Δ_W (%)
control	10.8 ± 0.9	2.1 ± 0.1	–
treated 3 min by HPEC	10.5 ± 0.7	2.3 ± 0.6	58 ± 8
treated 6 min by HPEC	10.3 ± 0.4	2.4 ± 0.4	75 ± 11
treated 6 min by the only $rGPM_{5.0}$	10.4 ± 0.7	2.5 ± 0.7	69 ± 15
immersed in water for 6 min	7.3 ± 0.8	3.3 ± 1.1	342 ± 43

[a]: The weight increment of the paper was calculated according to the equation, $\Delta_W = 100 \times (W_a - W_b) / W_b$, where, W_b and W_a are the weights of the paper sheets before and after treatment, respectively.

References

1. Suk, J. W., Piner, R. D., An, J. & Ruoff, R. S. Mechanical properties of monolayer graphene oxide. *ACS nano* **4**, 6557–6564 (2010).
2. Zhang, E. *et al*. Robust and thermo-response graphene–PNIPAm hybrid hydrogels reinforced by hectorite clay. *Carbon* **62**, 117–126 (2013).
3. Wang, Q. *et al*. High-water-content mouldable hydrogels by mixing clay and a dendritic molecular binder. *Nature* **463**, 339–343 (2010).
4. Calvet, D., Wong, J. Y. & Giasson, S. Rheological monitoring of polyacrylamide gelation: Importance of cross-link density and temperature. *Macromolecules* **37**, 7762–7771 (2004).
5. Guth, E. Theory of filler reinforcement. *J. Appl. Phys.* **16**, 20–25 (1945).
6. Lin, W. C. *et al*. Large strain and fracture properties of poly (dimethylacrylamide) / silica hybrid hydrogels. *Macromolecules* 2010, **43**, 2554–2563 (2010).
7. Li, Y. *et al*. In situ fabrication of Mn_3O_4 decorated graphene oxide as a synergistic catalyst for degradation of methylene blue. *Appl. Catal. B.* **162**, 268–274 (2015).
8. Gárcia-Fernández, I., Polo-López, M. I., Oller, I. & Fernández-Ibáñez, P. Bacteria and fungi inactivation using Fe^{3+}/sunlight, H_2O_2/sunlight and near neutral photo-Fenton: A comparative study. *Appl. Catal. B.* **121**, 20–29 (2012).
9. Segal, L., Creely, J., Martin J. & Conrad, C. M. An empirical method for estimating the degree of crystallinity of native cellulose using the X-ray diffractometer. *Text. Res. J.* **29**, 786–794 (1959).
